# Study on the correlation between total cerebral small vessel disease score and lacunar infarction

**DOI:** 10.3389/fneur.2025.1613080

**Published:** 2025-07-04

**Authors:** Sanqi Wang, Lijun Liu, Qingtang Meng, Xiaohang Su, Xinyu Zhou, Jijun Teng

**Affiliations:** Department of Neurology, The Affiliated Hospital of Qingdao University, Qingdao, China

**Keywords:** cerebral small vessel disease, total CSVD score, lacunar infarction, first-ever, recurrence

## Abstract

**Objective:**

To investigate the relationship between the total cerebral small vessel disease (CSVD) burden and the occurrence of first-ever and recurrent lacunar infarction (LI).

**Methods:**

This study included 271 patients with first-ever acute cerebral infarction hospitalized in the Department of Neurology, Affiliated Hospital of Qingdao University, between January 2019 and January 2024. The total CSVD score was calculated based on imaging findings. Patients were classified into LI and large-artery atherosclerosis (LAA) groups according to infarct size and large-vessel stenosis severity. The LI group was further subdivided into recurrence and non-recurrence subgroups. Clinical and imaging data were compared between groups. Logistic regression was used to identify risk factors for first-ever and recurrent LI, and the predictive value of the total CSVD score for LI recurrence was assessed using receiver operating characteristic (ROC) curve analysis.

**Results:**

The LI group comprised 153 patients (56.46%), and the LAA group included 118 patients (43.54%). Significant differences were observed in systolic blood pressure (SBP), diastolic blood pressure (DBP), uric acid (UA), albumin (ALB), triglycerides (TG), fibrinogen (FIB), total protein (TP), white matter hyperintensities (WMH), enlarged perivascular spaces (EPVS), lacunar, and total CSVD score between groups (*p* < 0.05). Logistic regression identified total CSVD score, WMH, EPVS, and UA as independent risk factors for first-ever LI, while FIB acted as a protective factor (*p* < 0.05). Among 140 LI patients, 28 experienced recurrence. Recurrent LI patients exhibited higher rates of smoking, WMH, EPVS, cerebral microbleeds (CMB), and total CSVD score compared to non-recurrent cases (*p* < 0.05). ROC analysis demonstrated that the total CSVD score predicted LI recurrence with the area under the curve (AUC) of 0.832.

**Conclusion:**

The total CSVD burden correlates with both first-ever and recurrent LIs. It is an independent risk factor for LI and may predict LI onset and progression.

## Introduction

Cerebral small vessel disease (CSVD) refers to structural or functional abnormalities in cerebral arterioles, capillaries, and venules that cause damage to brain parenchyma, leading to a spectrum of pathological, clinical, and imaging manifestations. These include cognitive impairment, dementia, stroke, mood disorders, and gait abnormalities (1). Typical neuroimaging features of CSVD encompass acute subcortical small infarcts, white matter hyperintensities (WMH), enlarged perivascular spaces (EPVS), cerebral microbleeds (CMB), lacunae, and cerebral atrophy (2). Isolated imaging markers may inadequately reflect the global burden of brain injury in CSVD, as these features frequently coexist and interact synergistically. Consequently, the concept of the total CSVD score has emerged in recent years to assess the cumulative impact of CSVD on cerebral function holistically.

Lacunar infarction (LI) arises from the occlusion of small perforating arteries in the cerebral hemispheres or brainstem, typically secondary to hypertensive arteriopathy or other vascular risk factors. These occlusions result in localized ischemic damage and neurological deficits, accounting for 20–30% of ischemic strokes (3, 4). While prior studies have linked individual CSVD imaging markers to ischemic stroke recurrence, the relationship between the total CSVD score and both first-ever and recurrent LI remains underexplored. This study aims to evaluate LI imaging characteristics systematically, identify risk factors for LI occurrence and recurrence, and inform strategies for secondary prevention.

## Methods

### Study population

**Figure 1:**
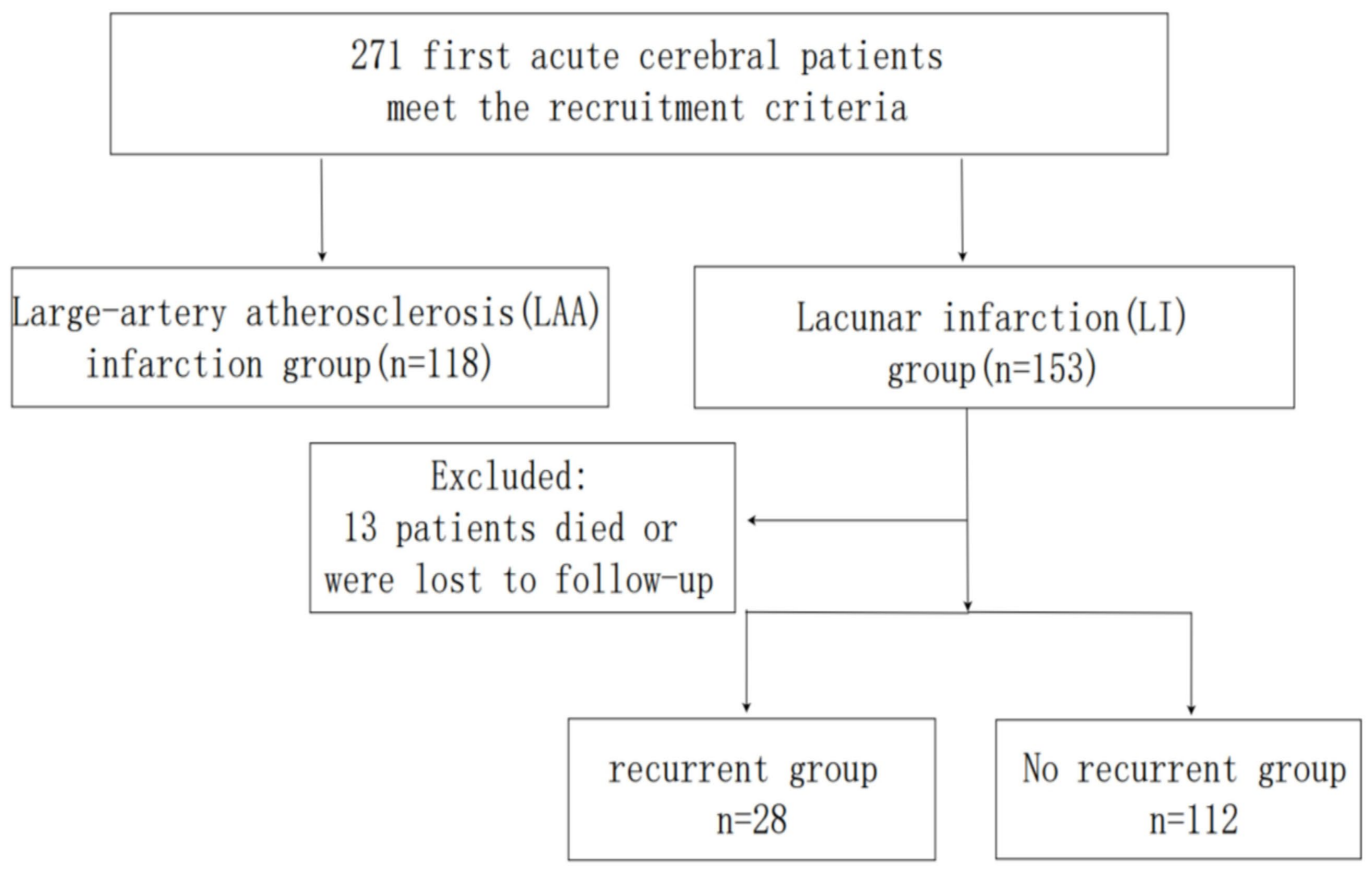
Schematic representation of the process for enrolling patients.

A total of 271 patients with first-ever cerebral infarction admitted to the Department of Neurology, Affiliated Hospital of Qingdao University, between January 2019 and January 2024 were retrospectively enrolled (Figure 1). Infarction subtypes were classified as LI or large-artery atherosclerosis (LAA). The first-ever cerebral infarction was defined as 1. Initial presentation of neurological deficits; 2. Brain magnetic resonance imaging (MRI) confirmed a single new ischemic lesion corresponding to clinical symptoms. Infarction subtypes were categorized according to the Trial of Org 10172 in Acute Stroke Treatment (TOAST) criteria. Ethical approval was obtained from the Ethics Committee of the Affiliated Hospital of Qingdao University and informed consent was obtained from all patient or their family members.

### Inclusion and exclusion criteria

#### Inclusion criteria

1. Diagnosis of first-ever LI or LAA confirmed by clinical and imaging assessments.
2. Age ≥18 years.
3. Admission and treatment within 7 days of symptom onset.
4. Availability of complete clinical data and follow-up records.

#### Exclusion criteria

1. Imaging or clinical evidence of other central nervous system pathologies (e.g., cerebral hemorrhage, subarachnoid hemorrhage, brain tumors, epilepsy).
2. Severe systemic comorbidities (e.g., cardiopulmonary insufficiency, severe arrhythmia, hepatic/renal dysfunction, active infection, thrombotic disorders, malignancy).
3. History of atrial fibrillation or congenital heart disease.
4. Prior thrombolytic therapy or endovascular intervention.
5. History of previous cerebral infarction.
6. Discordance between stenosis severity and infarction subtype:
  1. Patients with intracranial artery stenosis >50% but imaging findings consistent with LI.
  2. Patients with intracranial artery stenosis <50% but imaging findings consistent with LAA.
  3. Patients with intracranial artery stenosis <50% and imaging findings involving small artery perforator openings.

### Clinical data collection

Baseline demographic and clinical data were collected upon admission, including age, sex, average blood pressure at admission, smoking history, alcohol consumption, hypertension, diabetes, and other comorbidities. Laboratory parameters included fasting blood glucose (FBG), uric acid (UA), creatinine (Cr), albumin (ALB), total protein (TP), triglycerides (TG), total cholesterol (TC), low-density lipoprotein cholesterol (LDL-C), high-density lipoprotein cholesterol (HDL-C), apolipoprotein A1 (ApoA1), lipoprotein α, homocysteine (HCY), fibrinogen (FIB), and international normalized ratio (INR).

Neuroimaging studies were obtained for all patients, including brain MRI sequences (T_1_-weighted, T_2_-weighted, T_2_-fluid-attenuated inversion recovery [T_2_-FLAIR], susceptibility-weighted imaging [SWI], and diffusion-weighted imaging [DWI]), computed tomography angiography (CTA) of the brain and neck, and transthoracic echocardiography. A brain MRI was performed using a 3.0 T Signa HD superconducting scanner (GE Healthcare, USA). Intracranial and neck artery stenosis was assessed via CTA, and patients were classified into LI or LAA groups based on stenosis severity. LI recurrence was determined through telephone follow-up or hospital readmission records.

### Total CSVD score

Imaging manifestations of CSVD—WMH, EPVS, CMB, and lacunae were evaluated according to the 2024 international consensus criteria (2).

1. WMH: Defined as hyperintense lesions on T_2_-FLAIR sequences, symmetrically or asymmetrically distributed in periventricular or deep white matter. Severity was graded using the Fazekas scale:
  1. Periventricular WMH (PV-WMH): Fazekas score 3 (irregular hyperintensity extending into deep white matter).
  2. Deep WMH (D-WMH): Fazekas score 2–3 (confluent or extensive hyperintense lesions).
  3. A score of 1 point was assigned for PV-WMH = 3 or D-WMH ≥ 2.
2. EPVS: Fluid-filled spaces paralleling vessels, appearing linear (vessel-aligned) or round/oval (vessel-transverse) on MRI, with diameters <3 mm. Moderate-to-severe EPVS (≥11 lesions in the basal ganglia) was scored as 1 point.
3. CMB: Round/oval hypointense lesions (2–5 mm, ≤10 mm) on SWI or T_2_-weighted MRI in cortical/subcortical regions. The presence of ≥1 CMB was scored as 1 point.
4. Lacunae: Cavities (3–15 mm) with cerebrospinal fluid-like signal intensity (T_1_ hypointense, T_2_ hyperintense) in subcortical regions. The presence of ≥1 lacuna was scored as 1 point.

The total CSVD score (range: 0–4) was calculated using the validated scale by Staals et al., summing scores for WMH, EPVS, CMB, and lacunae (5).

### Statistical analysis

SPSS 25.0 software was used for statistical analysis. Continuous variables are expressed as mean ± standard deviation (normally distributed) or median [interquartile range] (non-normally distributed). Categorical variables are presented as percentages (%). The statistical data were analyzed by Pearson chi-square test or Fisher exact probability method. Nonnormal distribution data were tested using the Mann–Whitney U test or K-H test. Variables with *p* < 0.05 in univariate analysis, along with clinically relevant covariates, were entered into a binary logistic regression model to identify independent predictors of LI recurrence and associations with total CSVD score. Results are reported as odds ratios (OR) with 95% confidence interval (CI). The predictive value of the total CSVD score for LI recurrence was evaluated using receiver operating characteristic (ROC) curve analysis, with the area under the curve (AUC) calculated to quantify discrimination. Statistical significance was set at *p* < 0.05 (two-tailed).

## Results

A total of 271 patients with first-ever cerebral infarction were included in this study: 153 cases (56.46%) of LI and 118 cases (43.54%) of LAA. The LI group comprised 67.32% males, while the LAA group included 67.80% males. The demographic and clinical characteristics of both groups are summarized in Table 1. Significant differences were observed between the LI and LAA groups in systolic blood pressure (SBP), diastolic blood pressure (DBP), UA, ALB, TG, FIB, TP, WMH, EPVS, lacunae, and total CSVD score (*p* < 0.05).

**Table 1:** The baseline clinical data of the first-ever lacunar infarctions and large-artery atherosclerosis groups.

Characteristic	Lacunar infarction patients (*n* = 153)	Controls (*n* = 118)	*z*/*χ*^2^	*p*-values
Male, *n* (%)	103 (67.32%)	80 (67.80%)	χ2 = 0.007	0.934
Age (years)	61.00 (54.00–68.00)	63.00 (56.00–69.00)	*z* = −1.409	0.159
SBP (mmHg)	144.00 (134.00–156.00)	137.00 (128.00–148.25)	*z* = −3.271	0.001
DBP (mmHg)	82.00 (76.00–89.00)	77.00 (72.00–85.00)	*z* = −4.016	0.000
Diabetes, *n* (%)	38 (24.84%)	41 (34.75%)	*χ*^2^ = 3.167	0.075
Smoke, *n* (%)	74 (48.37%)	59 (50.00%)	*χ*^2^ = 0.071	0.790
Drink, *n* (%)	57 (37.25%)	53 (44.92%)	*χ*^2^ = 1.621	0.203
Laboratory index				
FBG (mmol/L)	5.14 (4.70–5.91)	5.49 (4.72–7.21)	*z* = −1.672	0.095
UA (μmol/L)	314.40 (281.00–373.05)	270.40 (214.35–325.33)	*z* = −4.915	0.000
Cr (μmol/L)	83.60 (72.95–93.25)	79.95 (70.30–87.73)	*z* = −1.794	0.073
ALB (g/L)	39.80 (37.70–41.68)	38.05 (35.28–39.90)	*z* = −4.815	0.000
TP (g/L)	63.78 (60.85–67.43)	61.66 (58.13–65.03)	*z* = −3.849	0.000
TG (mmol/L)	1.13 (0.81–1.49)	1.28 (0.96–1.76)	*z* = −2.497	0.013
TC (mmol/L)	4.19 (3.54–5.00)	4.24 (3.45–4.93)	*z* = −0.258	0.796
LDL-C (mmol/L)	2.54 (2.04–3.07)	2.50 (1.99–3.04)	*z* = −0.026	0.979
HDL-C (mmol/L)	1.17 (1.00–1.38)	1.08 (0.97–1.30)	*z* = −1.413	0.158
ApoA1 (g/L)	1.28 (1.15–1.42)	1.22 (1.07–1.46)	*z* = −1.892	0.059
Lipoprotein α (mmol/L)	178.00 (100.50–297.50)	186.50 (134.50–328.25)	*z* = −1.720	0.086
HCY (μmol/L)	11.50 (9.74–14.23)	10.94 (9.49–13.09)	*z* = −1.408	0.159
FIB (g/L)	2.58 (2.22–3.09)	2.97 (2.50–3.38)	*z* = −3.818	0.000
INR	11.00 (10.05–11.85)	11.15 (9.80–12.23)	*z* = −0.501	0.616
Imaging manifestations				
WMH score = 1, *n* (%)	71 (46.41%)	21 (17.80%)	*χ*^2^ = 24.316	0.000
CMB score = 1, *n* (%)	59 (38.56%)	34 (28.81%)	*χ*^2^ = 2.809	0.094
EPVS score = 1, *n* (%)	82 (53.60%)	26 (22.03%)	*χ*^2^ = 27.684	0.000
Lacunae score = 1, *n* (%)	91 (59.48%)	48 (40.68%)	*χ*^2^ = 9.424	0.002
Total CSVD score	2.00 (1.00–3.00)	1.00 (0.00–2.00)	*z* = −5.233	0.000

*p* < 0.05 = statistically significant result.

SBP, systolic blood pressure; DBP, diastolic blood pressure; FBG, fasting blood glucose; UA, uric acid; Cr, creatinine; ALB, albumin; TP, total protein; TG, triglycerides; TC, total cholesterol; LDL-C, low-density lipoprotein cholesterol; HDL-C, high-density lipoprotein cholesterol; ApoA1, apolipoprotein A1; HCY, homocysteine; FIB, fibrinogen; INR, international normalized ratio; WMH, white matter hyperintensities; CMB, cerebral microbleeds; EPVS, enlarged perivascular spaces; CSVD, cerebral small vessel disease.

Univariate and multivariate analyses were conducted to adjust for confounding factors and collinearity. A binary logistic regression model identified the total CSVD score (OR = 2.206, 95% CI: 1.630–2.985), WMH (OR = 4.929, 95% CI: 2.328–10.434), EPVS (OR = 4.103, 95% CI: 2.039–8.256), and UA (OR = 1.007, 95% CI: 1.003–1.012) as independent risk factors for first-ever LI, while FIB (OR = 0.309, 95% CI: 0.183–0.522) emerged as a protective factor (*p* < 0.05) (Table 2).

**Table 2:** Multivariate regression analysis of the first-ever lacunar infarctions and large-artery atherosclerosis groups.

Characteristic	*B*	OR (95% CI)	Adjust OR^*^ (95% CI)	*p*-values	*p*-values^*^
SBP	0.011	1.008 (0.995–1.022)	1.027 (0.996–1.060)	0.225	0.087
DBP	0.010	1.021 (0.987–1.056)	0.993 (0.949–1.040)	0.228	0.774
UA	0.007	1.007 (1.003–1.011)	1.007 (1.003–1.012)	0.000	0.001
ALB	0.052	1.074 (0.942–1.224)	1.054 (0.917–1.211)	0.285	0.367
TP	0.066	1.080 (0.991–1.177)	1.068 (0.975–1.170)	0.078	0.158
FIB	−1.174	0.294 (0.175–0.496)	0.309 (0.183–0.522)	0.000	0.000
WMH	1.595	4.230 (2.049–8.734)	4.929 (2.328–10.434)	0.000	0.000
EPVS	1.412	3.434 (1.781–6.621)	4.103 (2.039–8.256)	0.000	0.000
Lacunae	0.362	1.446 (0.767–2.726)	1.436 (0.757–2.723)	0.255	0.268
Total CSVD score	0.791	2.128 (1.597–2.836)	2.206 (1.630–2.985)	0.000	0.000

*p* < 0.05 = statistically significant result. Adjust OR^*^: adjustment for age, gender.

SBP, systolic blood pressure; DBP, diastolic blood pressure; UA, uric acid; ALB, albumin; TP, total protein; FIB, fibrinogen; WMH, white matter hyperintensities; EPVS, enlarged perivascular spaces; CSVD, cerebral small vessel disease; OR, odds ratios; CI, confidence interval.

Among 153 LI patients, 13 were lost to follow-up (due to death or contact information changes), leaving 140 patients for recurrence analysis. Of these, 28 patients (20.0%) experienced recurrence, while 112 (80.0%) remained recurrence-free. Clinical and imaging characteristics of the recurrence and non-recurrence groups are detailed in Table 3. Significant differences were observed in smoking history, WMH, EPVS, CMB, lacunae, total CSVD score, FIB, and lipoprotein α (*p* < 0.05).

**Table 3:** The baseline clinical data of the non-recurrence and recurrence groups of the lacunar infarction patients.

Characteristic	Recurrent group (*n* = 28)	No recurrent group (*n* = 112)	*z*/*χ*^2^	*p*-values
Male, *n* (%)	21 (75.00%)	76 (67.86%)	*χ*^2^ = 0.537	0.464
Age (years)	63.00 (55.00–70.75)	61.00 (54.00–67.00)	*z* = −1.069	0.285
SBP (mmHg)	141.00 (133.00–154.50)	144.00 (134.00–155.50)	*z* = −0.300	0.764
DBP (mmHg)	85.50 (75.25–93.00)	81.00 (75.00–88.00)	*z* = −0.983	0.326
Diabetes, *n* (%)	21 (75.00%)	87 (77.68%)	*χ*^2^ = 0.091	0.763
Smoke, *n* (%)	21 (75.00%)	50 (44.64%)	*χ*^2^ = 8.259	0.004
Drink, *n* (%)	9 (32.14%)	42 (37.50%)	*χ*^2^ = 0.278	0.598
Laboratory index				
FBG (mmol/L)	4.99 (4.55–6.96)	5.14 (4.71–5.79)	*z* = −0.425	0.671
UA (μmol/L)	291.00 (258.85–335.90)	313.80 (280.93–372.05)	*z* = −0.891	0.373
Cr (μmol/L)	75.85 (60.93–92.78)	84.10 (73.98–92.38)	*z* = −1.633	0.102
ALB (g/L)	39.11 (37.71–42.57)	39.95 (37.60–41.69)	*z* = −0.320	0.749
TP (g/L)	63.59 (60.30–70.02)	63.74 (60.93–66.60)	*z* = −0.406	0.684
TG (mmol/L)	1.24 (0.89–1.80)	1.28 (1.01–1.68)	*z* = −0.610	0.542
TC (mmol/L)	4.31 (3.13–5.57)	4.18 (3.54–4.97)	*z* = −0.049	0.961
LDL-C (mmol/L)	2.69 (1.62–3.37)	2.44 (2.05–3.06)	*z* = −0.208	0.835
HDL-C (mmol/L)	1.19 (1.03–1.34)	1.17 (0.96–1.41)	*z* = −0.503	0.615
ApoA1 (g/L)	1.31 (1.23–1.39)	1.26 (1.13–1.43)	*z* = −0.972	0.331
Lipoprotein α (mmol/L)	121.50 (54.00–276.25)	184.00 (109.50–310.00)	*z* = −2.170	0.030
HCY (μmol/L)	11.89 (10.09–13.47)	11.17 (9.63–14.87)	*z* = −0.563	0.574
FIB (g/L)	2.93 (2.52–3.21)	2.47 (2.10–3.04)	*z* = −2.420	0.016
INR	11.05 (9.90–12.08)	11.10 (10.30–11.80)	*z* = −0.023	0.981
Imaging manifestations				
WMH score = 1, *n* (%)	22 (78.57%)	38 (33.93%)	*χ*^2^ = 18.229	0.000
CMB score = 1, *n* (%)	20 (71.43%)	31 (27.68%)	*χ*^2^ = 18.514	0.000
EPVS score = 1, *n* (%)	25 (89.29%)	47 (41.96%)	*χ*^2^ = 20.081	0.000
Lacunae score = 1, *n* (%)	23 (82.14%)	56 (50.00%)	*χ*^2^ = 9.413	0.002
Total CSVD score	4.00 (3.00–4.00)	1.00 (1.00–2.00)	*z* = −5.553	0.000

*p* < 0.05 = statistically significant result.

SBP, systolic blood pressure; DBP, diastolic blood pressure; FBG, fasting blood glucose; UA, uric acid; Cr, creatinine; ALB, albumin; TP, total protein; TG, triglycerides; TC, total cholesterol; LDL-C, low-density lipoprotein cholesterol; HDL-C, high-density lipoprotein cholesterol; ApoA1, apolipoprotein A1; HCY, homocysteine; FIB, fibrinogen; INR, international normalized ratio; WMH, white matter hyperintensities; CMB, cerebral microbleeds; EPVS, enlarged perivascular spaces; CSVD, cerebral small vessel disease.

Multivariate regression analysis adjusted for confounders revealed smoking (OR = 13.265, 95% CI: 1.907–92.295), WMH (OR = 3.607, 95% CI: 1.098–11.852), EPVS (OR = 8.511, 95% CI: 1.976–36.648), and total CSVD score (OR = 3.276, 95% CI: 2.019–5.315) as independent predictors of LI recurrence (*p* < 0.05) (Table 4).

**Table 4:** Multivariate regression analysis of the non-recurrence and recurrence groups of the lacunar infarction patients.

Characteristic	*B*	OR (95% CI)	Adjust OR^*^ (95% CI)	*p*-values	*p*-values^*^
Smoke	2.585	4.412 (1.455–13.378)	13.265 (1.907–92.295)	0.009	0.009
WMH	1.283	3.333 (1.060–10.486)	3.607 (1.098–11.852)	0.040	0.035
CMB	3.696	3.696 (1.185–11.530)	3.832 (1.176–12.485)	0.024	0.026
EPVS	2.141	6.872 (1.730–27.290)	8.511 (1.976–36.648)	0.006	0.004
Lacunae	0.133	1.266 (0.350–4.579)	1.143 (0.306–4.262)	0.719	0.843
Total CSVD score	−4.907	3.179 (1.992–5.073)	3.276 (2.019–5.315)	0.000	0.000

*p* < 0.05 = statistically significant result. Adjust OR^*^: adjustment for age, gender.

WMH, white matter hyperintensities; CMB, cerebral microbleeds; EPVS, enlarged perivascular spaces; CSVD, cerebral small vessel disease; OR, odds ratios; CI, confidence interval.

**Figure 2:**
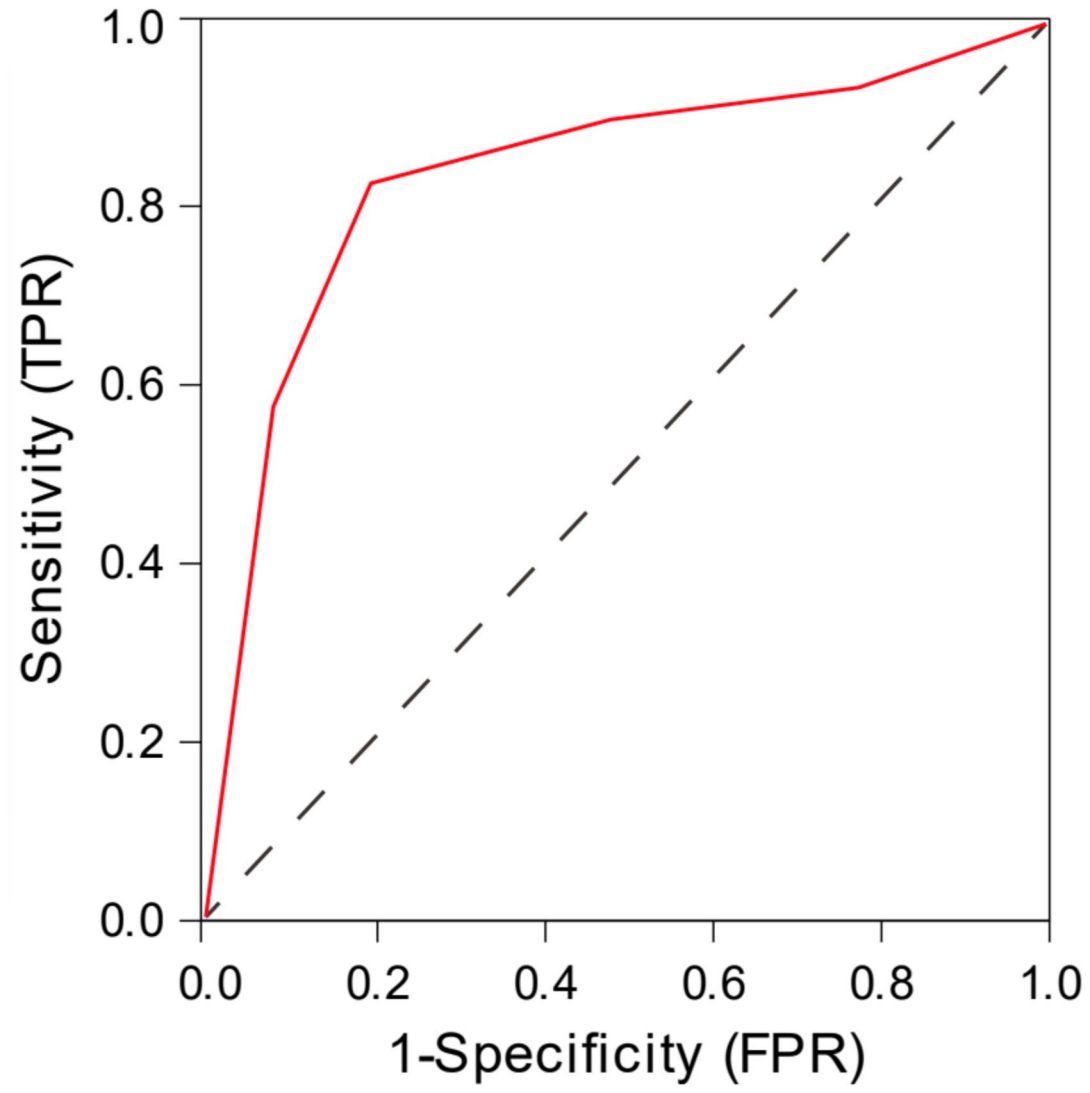
The ROC curve analysis of total CSVD score in predicting the recurrence of lacunar infarction.

The ROC curve analysis demonstrated that the total CSVD score predicted LI recurrence with an AUC of 0.832 (95% CI: 0.737–0.928). The optimal cutoff value was 2.5, yielding a sensitivity of 82% and a specificity of 80% (Figure 2).

## Discussion

### Analysis of risk factors for the first LI

This study first explored risk factors for first-ever LI by comparing clinical and imaging markers between LI and LAA groups. Our results identified UA, WMH, EPVS, and total CSVD score as independent risk factors for first-ever LI, while FIB emerged as a protective factor. UA, a purine metabolite derived from adenosine/guanine breakdown, is implicated in atherosclerosis, particularly small-vessel pathology. While UA initially acts as an antioxidant by scavenging free radicals, elevated levels paradoxically promote oxidative stress, endothelial dysfunction, and chronic inflammation (6). Hyperuricemia exacerbates vascular injury through UA crystal deposition in arteriolar walls, impairing nitric oxide (NO) synthesis and endothelial homeostasis (6, 7). These mechanisms align with findings linking UA to EPVS severity (8) and WMH progression in LI patients (9). Furthermore, UA correlates with cognitive decline in LI, highlighting its role as a biomarker for CSVD burden (10).

Contrary to expectations, our study identified FIB as a protective factor against LI compared to LAA. Elevated FIB typically reflects atherosclerosis progression by promoting vascular wall lipid deposition and thrombosis (11). However, LI pathogenesis appears more driven by inflammatory pathways, whereas LAA is strongly associated with thrombotic mechanisms. This distinction is supported by Alvarez-Perez et al., who reported a higher erythrocyte sedimentation rate (ESR) in LI (inflammatory dominance) versus elevated D-dimer in LAA (thrombosis dominance) (12). Thus, FIB’s protective role in LI may reflect compensatory anti-inflammatory effects or divergent pathophysiological pathways between infarction subtypes.

### The correlation between CSVD imaging findings and the first-ever LI

As a key neuroimaging manifestation of CSVD, WMH are believed to result from chronic vascular ischemic injury. In contrast, LI represents an acute ischemic manifestation of CSVD, typically caused by hypertension-induced arterial flow disturbances, with both pathologies sharing common vascular risk factors. Consistent with our findings, Xu et al. demonstrated that WMH progression exhibits a time-dependent association with LI incidence, particularly within the initial 2-year period post-stroke, and may serve as an independent risk factor for LI development (13). Notably, despite methodological differences in follow-up duration between studies, this correlation remains statistically significant.

The perivascular space functions as a critical cerebral clearance pathway for metabolic waste, maintaining fluid homeostasis. EPVS indicates compromised waste elimination and fluid dynamics, manifesting as impaired glymphatic transport and reduced clearance capacity. Experimental evidence suggests that glymphatic stasis may induce vascular distension and tissue edema, potentially exacerbating endothelial ischemic injury (14). Supporting this mechanism, a prospective cohort study of 50 LI patients revealed elevated cerebral extracellular free water content secondary to impaired clearance mechanisms and endothelial dysfunction, collectively aggravating CSVD pathology (15).

Current research consensus emphasizes the superior prognostic value of total CSVD score over individual imaging biomarkers for stroke risk stratification and outcome prediction. In a retrospective analysis of 396 first-ever LI patients, Tang et al. demonstrated significantly higher composite CSVD burden than MRI-normal controls, suggesting that comprehensive assessment better reflects cerebral microvascular ischemic damage (16). Furthermore, elevated CSVD burden not only increases LI susceptibility but also correlates with post-stroke cognitive decline, as evidenced by population studies (17).

### Analysis of risk factors for recurrent LI

After adjusting for confounders, including age and gender, the results demonstrated that smoking, WMH, EPVS, and total CSVD score emerged as independent risk factors for LI recurrence.

The established role of smoking as an independent risk factor for primary stroke (18) is reinforced by our findings in recurrent events. Bonita et al. documented a sixfold increased stroke risk among smokers, with a distinct dose–response relationship between cigarette consumption and stroke incidence. Remarkably, even passive smoke exposure conferred a 1.82-fold elevated stroke risk (19). This correlation may be mediated through smoking’s association with greater CSVD burden observed in first-ever LI patients (16).

Multivariate analysis newly identifies smoking as a significant contributor to stroke recurrence, potentially through multiple pathophysiological mechanisms. Tobacco-derived neurotoxins, including nicotine and carbon monoxide, induce direct endothelial damage while promoting platelet aggregation and blood hyperviscosity. These effects synergistically drive systemic chronic inflammation, exacerbating microvascular dysfunction and cerebral hypoperfusion processes that collectively heighten LI recurrence risk. Clinical evidence substantiates that smoking cessation reduces recurrence risk by 50%, with maximal benefits accruing within the initial 6-month abstinence period (20).

### The correlation between CSVD imaging findings and recurrent LI

Recurrent ischemic stroke in CSVD typically manifests with more pronounced imaging abnormalities. Consistent with our findings, Park et al. demonstrated that severe white matter hyperintensities WMH reflect advanced CSVD pathology and exhibit strong predictive value for stroke recurrence risk, establishing WMH as a key imaging biomarker for vascular event recurrence (21). This conclusion was confirmed in the study of Ryu et al., who identified a quantitative association between WMH volume and ischemic stroke recurrence (22).

Emerging evidence suggests topographic specificity in WMH-related stroke risk. Notably, Liu et al. reported differential recurrence risks between PV-WMH and D-WMH, with PV-WMH demonstrating superior predictive capacity. This dichotomy likely reflects distinct pathogenic mechanisms: D-WMH primarily associates with hypertensive arteriopathy characterized by arteriolar hyalinosis, wall thickening, and luminal stenosis, whereas PV-WMH may originate from hemodynamic impairment in watershed regions. Remarkably, PV-WMH has been independently associated with recurrent events in acute ischemic stroke populations (23).

CMB constitutes another critical prognostic marker, conferring increased risks for recurrent ischemic stroke, intracerebral hemorrhage, and mortality. Shoamanesh et al. quantified this relationship, revealing that CMB presence elevates ischemic stroke recurrence risk and predicts worse composite vascular outcomes (24). Notably, CMB topography modulates the recurrence probability. Both lobar and deep CMBs demonstrate higher recurrence rates than CMB-negative cases, with combined lobar and deep CMBs exhibiting the highest recurrence risk (25, 26).

Our findings corroborate existing evidence regarding EPVS. Lau et al. established dose-dependent relationships, showing basal ganglia EPVS >20 conferred 1.8-fold and 2.6-fold increased risks for ischemic and hemorrhagic stroke recurrence, respectively, compared to <11 EPVS (27). Furthermore, basal ganglia EPVS burden independently predicts poor functional outcomes post-ischemic stroke (28).

The composite CSVD burden score demonstrates particular clinical utility. Multicenter prospective data from anticoagulated atrial fibrillation patients with prior stroke/transient ischemic attack (TIA) revealed that CSVD severity significantly correlated with recurrent ischemic stroke risk during follow-up (29). This underscores the necessity for CSVD-adapted anticoagulation strategies in cardioembolic stroke. Contrarily, Tian et al. failed to identify independent associations between CSVD severity and recurrence, instead proposing combined infarct number as a superior predictor (30). This discrepancy highlights the need for standardized CSVD assessment protocols.

The total CSVD score demonstrates critical prognostic value in both the first-ever and recurrent LI. Notably, as a distinct ischemic stroke subtype, LI exhibits early progressive characteristics, necessitating differential analysis from LAA mechanisms to enable targeted preventive strategies for CSVD progression mitigation and LI risk reduction.

Mechanistically, the total CSVD score modulates stroke pathogenesis through cerebral hypoperfusion and blood pressure variability, culminating in blood–brain barrier disruption, compromised vascular integrity, and cumulative parenchymal damage. Notably, key neuroimaging markers—WMH, EPVS, CMB, and lacunes—collectively mediate this pathological cascade. With the accumulation of age and time, CSVD progression impairs cerebrovascular autoregulation and collateral compensation capacity, ultimately predisposing to cerebral hypoperfusion and adverse clinical outcomes.

Our study also has many shortcomings: 1. Retrospective design limitations: Variable follow-up durations compromised temporal resolution. Notably, multivariate regression failed to establish significant associations between lacunar and LI occurrence/recurrence. In the future, more prospective studies are needed to seek more accurate conclusions. 2. Sample characteristics: Single-center recruitment with a limited sample size introduced selection bias, restricting generalizability. 3. Collinearity challenges: Inherent correlations between CSVD score components and individual biomarkers precluded combined modeling, necessitating separate analyses that compromised result stability. 4. Imaging granularity: The study did not subdivide a single imaging marker according to location, size, number, etc. Future studies can appropriately expand the CSVD scoring criteria and further refine the prediction of each imaging marker for the occurrence and development of stroke. 5. Due to the limitations of this study, we did not explore the relationship between genetic factors and lacunar infarction, in order to expect more extensive research in the future to explore the correlation between the two.

These problems need to be further solved by follow-up studies.

## Conclusion

The total CSVD score demonstrates significant associations with both incident and recurrent LI, establishing itself as an independent predictor for these clinical endpoints. Notably, this biomarker holds particular promise for early risk stratification in clinical practice, enabling preemptive evaluation of LI susceptibility before symptom manifestation. Additionally, its predictive power derives from quantifying cumulative microvascular damage underlying cerebral hypoperfusion and blood–brain barrier failure.

## Data Availability

The original contributions presented in the study are included in the article/supplementary material, further inquiries can be directed to the corresponding author.
